# Efficacy and Safety of Fecal Microbiota Transplantation for Clearance of Multidrug-Resistant Organisms under Multiple Comorbidities: A Prospective Comparative Trial

**DOI:** 10.3390/biomedicines10102404

**Published:** 2022-09-26

**Authors:** Jongbeom Shin, Jung-Hwan Lee, Soo-Hyun Park, Boram Cha, Kye Sook Kwon, Hyungkil Kim, Yong Woon Shin

**Affiliations:** 1 Division of Gastroenterology, Department of Internal Medicine, Inha University Hospital, Inha University School of Medicine, Incheon 22332, Korea; 2 Department of Hospital Medicine, Inha University Hospital, Inha University School of Medicine, Incheon 22332, Korea

**Keywords:** multidrug resistance, fecal microbiota transplantation, dysbiosis, carbapenem-resistant *Enterobacteriaceae*, vancomycin-resistant enterococci

## Abstract

Fecal microbiota transplantation (FMT) could decolonize multidrug-resistant organisms. We investigated FMT effectiveness and safety in the eradication of carbapenem-resistant *Enterobacteriaceae* (CRE) and vancomycin-resistant enterococci (VRE) intestinal colonization. A prospective non-randomized comparative study was performed with 48 patients. FMT material (60 g) was obtained from a healthy donor, frozen, and administered via endoscopy. The primary endpoint was 1-month decolonization, and secondary endpoints were 3-month decolonization and adverse events. Microbiota analysis of fecal samples was performed using 16S rRNA sequencing. Intention-to-treat analysis revealed overall negative conversion between the FMT and control groups at 1 (26% vs. 10%, *p* = 0.264) and 3 (52% vs. 24%, *p* = 0.049) months. The 1-month and 3-month CRE clearance did not differ significantly by group (36% vs. 10%, *p* = 0.341; and 71% vs. 30%, *p* = 0.095, respectively). Among patients with VRE, FMT was ineffective for 1-month or 3-month negative conversion (13% vs. 9%, *p* > 0.999; and 36% vs. 18%, *p* = 0.658, respectively) However, cumulative overall negative-conversion rate was significantly higher in the FMT group (*p* = 0.037). *Enterococcus* abundance in patients with VRE significantly decreased following FMT. FMT may be effective at decolonizing multidrug-resistant organisms in the intestinal tract.

## 1. Introduction

Infections caused by multidrug-resistant organisms (MDROs) are a major challenge to healthcare systems worldwide [1,2]. MDRO carriage has been a concern in hospital-acquired infections because MDRO infections can be fatal [3]. Among these organisms, carbapenem-resistant *Enterobacteriaceae* (CRE) and vancomycin-resistant enterococci (VRE) are considered a serious problem because antibiotics are ineffective against them. CRE can spread in healthcare settings and have caused infections with mortality rates of 40% to 50% in the USA [4]. South Korea reported an increased incidence of CRE carriers from 5717 in 2017 to 14,205 in 2019 [5]. The prevalence of VRE has also increased worldwide. In a US study, up to 80% of *Enterococcus faecium* isolates were demonstrated to be vancomycin resistant [6]. Furthermore, VRE constituted 36.5% of the enterococci isolated from domestic medical institutions in South Korea [7]. Moreover, VRE or CRE colonization in feces causes economic burden and additional restrictions such as isolation for the prevention of transmission [8].

Recently, fecal microbiota transplantation (FMT) has been reported to be a possible option for the decolonization of CRE and VRE [9,10,11,12]. The clearance of CRE and VRE by FMT has been reported to be approximately 60–70% in prospective studies [11,13]. However, most of previous studies included a small number of patients and were not well-designed, lacking a control group [10,12,14,15,16,17,18]. In addition, previous studies employing microbiome analysis could not determine the mechanism underlying MDRO decolonization [9,10,12,16]. Many studies on FMT for MDRO decolonization also did not evaluate the safety of this approach [10]. Patients with MDRO colonization tend to have multiple comorbidities and require long-term use of antibiotics that may cause CRE or VRE colonization. The safety of FMT for MDRO decolonization in these patients should be verified.

In 2014, our institute established the Microbiome Center, which focuses on the development of effective FMT [19]. We prospectively studied the application of FMT for the decolonization of MDROs, including CRE and VRE, from 2019. The aim of this study was to determine, via microbiome analysis, whether FMT is effective and safe for eradicating intestinal colonization by MDROs.

## 2. Materials and Methods

### 2.1. Patient Recruitment

All patients and donors agreed to participate in a clinical trial of FMT for eliminating intestinal colonization by MDROs at Inha University Hospital, Incheon, Korea. We enrolled patients aged ≥18 years who were diagnosed with CRE or VRE colonization via stool culture with at least two consecutive rectal swabs. We performed a prospective non-randomized comparative study from November 2019 to November 2021, excluding patients with known immunosuppression (HIV infection or steroid therapy); acute infectious diseases (except *Clostridioides difficile* colitis); pregnant or breastfeeding women; patients with structural abnormalities in the digestive tract such as intestinal obstruction, severe ileus, or perforation; patients with critical illness requiring intensive care unit admission; and patients with ongoing therapy with antibiotics that could lead to the development of MDRO, such as glycopeptides and carbapenems. Ongoing antibiotic therapy was not discontinued prior to FMT, but antibiotics that could affect the development of MDRO were avoided. For example, glycopeptides, including vancomycin or teicoplanin, were not used in patients with VRE, and carbapenems were not used in patients with CRE. If these antibiotics were used, the patients were withdrawn from the study. In the control group, no medications or procedures that might affect MDRO clearance were used [20]. Allocation to the FMT or control group was based on participant preference.

### 2.2. Donor

The stool donor was a healthy 20-year-old man without any underlying disease, who was not on any medication. We confirmed his clinical status by stool and serologic tests, including a COVID-19 test. The condition of the donor was regularly checked via laboratory and stool tests. He also underwent physical examination and blood tests (complete blood count, blood glucose, electrolytes, inflammatory markers, and liver function tests) to check for gastrointestinal, metabolic, or neurological disorders. Serology screening for HIV, syphilis, and hepatitis A, B, and C was also performed. Stool PCR analysis was conducted for pathogenic bacteria (*Shigella* spp., *Salmonella* spp., *Campylobacter* spp., *Yersinia* spp., and toxin-producing *C. difficile*) and viruses (astrovirus, enteric adenovirus, rotavirus, and norovirus). Stool samples were also examined for ova and parasites. All tests and examinations yielded negative results.

### 2.3. Fecal Sample Collection, Preparation, and Administration

Prior to FMT, each patient’s condition, including diseases requiring hospitalization and treatment, comorbidities, and MDRO colonization duration, was assessed. Stool samples were collected for MDRO culture tests and biochemical analyses. We used frozen fecal microbiota stored in a stool bank at −80 °C; the sample was warmed to room temperature before transplantation. On the day of transplantation, the fecal samples were weighed, and 60 g portions were mixed with 40 mL of sterile saline (0.9% NaCl), filtered through a 110 cm × 10 cm non-woven swab, drawn into 50 mL sterile syringes, sealed, and stored immediately in the stool bank until transplantation. Bowel preparation before FMT was not essential and the procedure was performed according to the patient’s condition. Each transplant was administered to the distal duodenum via a gastro-endoscope or to the cecum or colon via colonoscopy, through the working channel of the endoscope, which was then flushed with 50 mL of sterile saline.

### 2.4. Outcome

The primary endpoint was 1-month CRE- or VRE-negative conversion. The secondary endpoints were 3-month CRE- or VRE-negative conversion and adverse events. All patients had control swabs (culture) on days 7, 14, 21, and 28, and 3 months after the FMT, if possible. Negative conversion means at least 3 consecutive negative results at rectal swab. The time of negative conversion was defined as the first result of 3 consecutive negative results. Adverse events were recorded during and after FMT. All patients were followed up for 3 months.

### 2.5. Stool Bacterial Analysis for Metagenomics Analysis

The patients’ feces (about 2−5 mL) were collected in a stool carrier and stored in a refrigerator at −80 °C. We sent these fecal samples, and following processing to 16S rRNA gene amplicon sequencing was performed at Macrogen Inc. (Seoul, Korea).

Metagenomic DNA was extracted with a QIAamp stool kit, and amplification of the V3-V4 region of the bacterial 16S rRNA gene was conducted using barcoded universal primers. PicoGreen was used to pool and normalize the amplified products. Quantity and fragment size of extracted DNA were assessed using Agilent Technologies 2100 Bioanalyzer using a DNA 1000 chip (Santa Clara, CA, USA). Sequencing was carried out using a MiSeq sequencer on an Illumina platform) according to the manufacturer’s specifications. By assembling paired-end reads created by sequencing both directions of the library, the original library and single long reads were obtained. The program used in this process was FLASH (version 1.2.11) [21]. For precise operational taxonomic unit (OTU) analysis, the data containing sequence errors were removed. Reads that contained an ambiguous base and chimeric sequence were removed because these implied sequencing errors.

Among the assembled reads, reads shorter than 400 bp or longer than 500 bp were removed. After this process, clustering was performed based on the sequence similarity. The OTUs of the remaining reads were created using a cluster cut-off value of 97% in the CD-HIT-OTU program [22].

QIIME and the Phyloseq R package were used for OTU analysis and taxonomy information [23]. The major sequence of each OTU was referred to the NCBI 16S DB, and taxonomy information was obtained using BLAST+ (v.2.9.0) [24]. To confirm the diversity and evenness of the microbial community, Shannon and Chao1 indices were calculated [25]. Beta-diversity (diversity among samples within a group) was calculated based on the unweighted UniFrac distance and Bray–Curtis distance. Genetic relationships among samples were visualized using principal coordinate analysis (PCoA).

To compare the relative abundance of microbial communities in the pre- and post-FMT groups, a group average bar graph was created for each genus representing at least 1% of at least one group.

### 2.6. Ethical Considerations

This clinical study was approved by the ethics committee of Inha University Hospital (INHAUH 2018-08-015). All patients and donors were informed of the benefits and potential risks of standardized FMT and laboratory screening. Written informed consent was obtained from all participants. This trial is registered with the Clinical Research Information Service of Korea (https://cris.nih.go.kr/; KCT0004423 (accessed on 18 September 2022).

### 2.7. Sample Size

We assumed a spontaneous loss of detectable VRE or CRE carriage in 20% of the patients at 1 month in the control group and a further absolute reduction by 60% in the intervention group [10,26,27,28]. Assuming an 80% power at a two-sided significance level of 10% and an expected 10% rate of loss to follow-up, a targeted sample size of 50 participants was calculated (α = 0.05, 1 − β = 0.80).

### 2.8. Statistical Analysis

Considering the loss of follow-up participants, the primary outcome was analyzed according to the intention-to-treat (ITT) principle. The ITT population comprised all patients enrolled. Missing data for the primary outcome were imputed according to the last record of MDRO colonization. If the last record of MDRO colonization was positive, then it was marked as positive, and if it was negative, then it was documented as continuously negative. We also performed a per-protocol analysis (defined after the end of the study but before analysis) where patients were analyzed according to the treatment received. The per-protocol population consisted of patients who fulfilled the following criteria: patients with non-missing data and achieving the primary outcome or secondary outcome. Univariate logistic regression with group assignment as a predictor variable and decolonization as the outcome variable was performed to calculate the odds ratio (OR) and 95% confidence interval (CI). Fisher’s exact test was used to analyze categorical variables. The differences in microbiota between groups were analyzed using the Wilcoxon rank-sum test for two independent samples or the Wilcoxon signed-rank test for two related samples or matched samples. Data analysis was performed using R (R Foundation, Vienna, Austria; https://www.r-project.org (accessed on 18 September 2022) or IBM SPSS 19 (IBM Corp., Armonk, NY, USA) software. Permutational multivariate analysis of variance (PERMANOVA) was used to determine the significance of the PCoA plot results. Statistical significance was set at *p* < 0.05. Linear discriminant analysis effect size (LEfSe) was determined and visualized to confirm the difference in relative abundance between groups.

## 3. Results

### 3.1. Patient Characteristics

**Figure 1:**
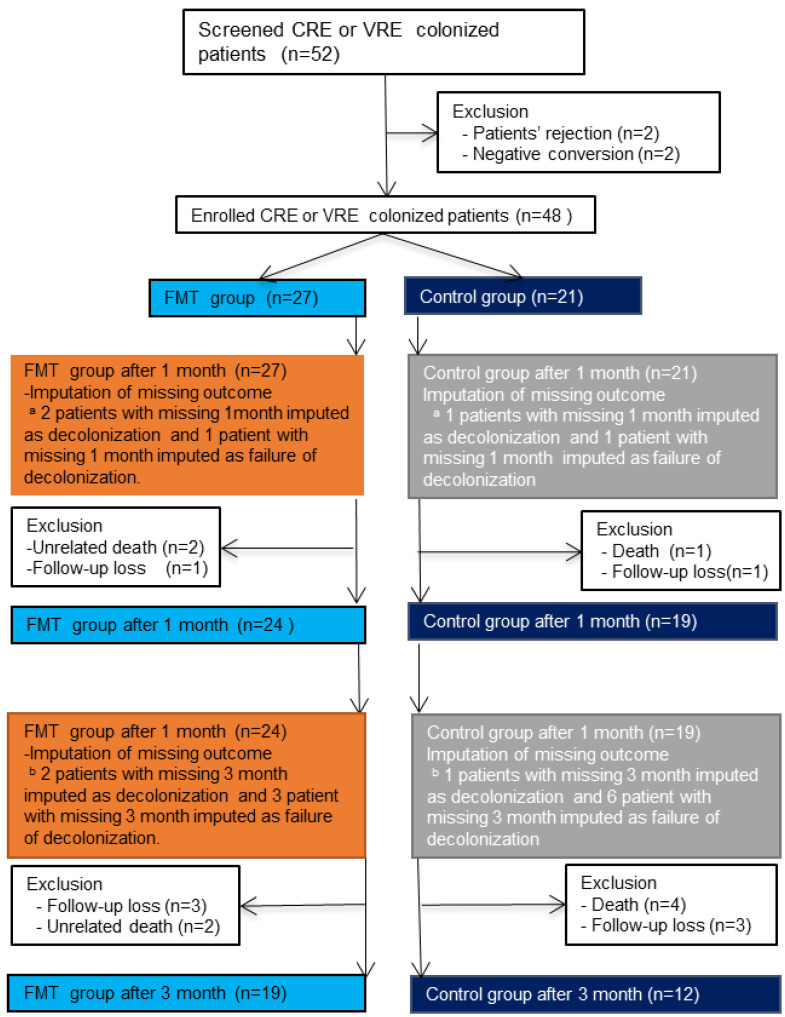
Flow chart of patient enrolment. **a:** imputation of missing outcome from enrollment to 1 month. **b:** imputation of missing outcome from 1 month to 3 month.

Fifty-two patients with CRE and VRE colonization were screened from November 2019 to November 2021, and four patients were excluded before enrolment (Figure 1). Thus, 48 patients with CRE and VRE colonization were enrolled, with 27 in the FMT group and 21 in the control group. Among them, 24 patients in the FMT group and 19 patients in the control group completed the 1-month follow-up; and 19 patients in the FMT group and 12 patients in the control group completed the 3-month follow-up. The baseline clinical characteristics of the FMT and control groups are shown in Table 1. Patients in the FMT group were significantly younger than those in the control group. However, the other variables, such as multidrug resistance, disease on admission, and the Charlson Comorbidity Index, did not differ significantly between the two groups. Types of using antibiotics between FMT group and control group was also not different between both groups (Supplementary Table S1). The 1-month and 3-month MDRO-negative conversion was not significantly associated with age in all patients or in the FMT group (Supplementary Tables S2 and S3).

Next, we performed FMT via colonoscopy in approximately half of the patients in the FMT group who underwent bowel preparation before FMT. Twelve patients had contraindications to bowel preparation because of medical conditions such as chronic kidney disease and heart failure. Patients in the FMT group received FMT either through duodenoscopy or sigmoidoscopy (as a 20 g or 30 g infusion, respectively). One patient had a colonic stricture so FMT was administered via duodenoscopy, without bowel preparation. Four patients had severe *C. difficile*-associated diarrhea; thus, FMT was administered into the cecum via colonoscopy, without bowel preparation.

### 3.2. Withdrawal, Loss to Follow-Up, and Missing Data

One patient in the FMT group died because of food aspiration. The patient was infected with VRE and CRE. In this patient, CRE switched to negative conversion at 2 weeks after FMT. In the ITT analysis, we assumed that negative conversion was achieved for CRE 1 month after FMT. Another patient died of a sudden heart attack 1 week after FMT. The Institutional Review Boards assessed that the cause of death was not directly related to FMT. This patient did not achieve negative conversion for VRE after 1 week, and thus, the primary endpoint was considered not achieved.

Within 3 months, one patient in the FMT group died owing to a sudden decrease in blood pressure during hemodialysis. patient in the intervention group died owing to pneumonia 6 weeks after FMT. Both patients did not achieve the negative conversion of MDROs (one CRE, and the other VRE and CRE) or the secondary outcomes. Moreover, four patients in the control group died.

One patient was lost to follow-up within 1 month of enrolment in both the FMT and the control groups. Three patients in the FMT group and three patients in the control group were lost to follow-up within 3 months. There were no significant differences between groups in unrelated death and loss-to-follow-up within 1 month (*p* = 0.856 and *p* = 0.563, respectively) or within 3 months (*p* = 0.428 and *p* > 0.999, respectively).

### 3.3. Clearance of Multidrug-Resistant Organisms

**Figure 2:**
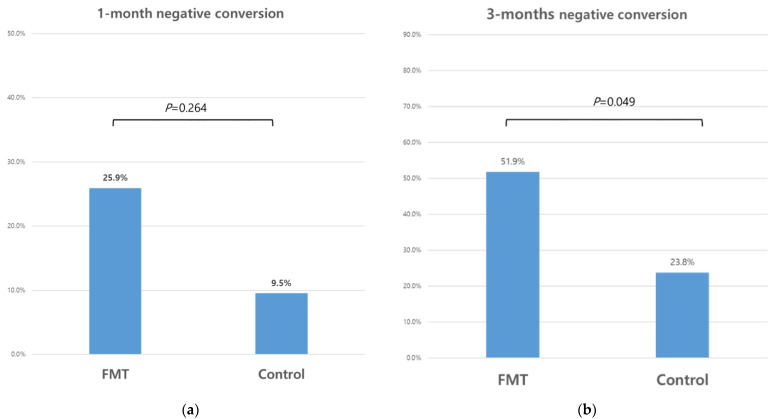
Multidrug-resistant organism (MDRO) clearance rate according to intention-to-treat analysis following fecal microbiota transplantation (FMT). (**a**) 1-month negative-conversion rate (**b**) 3-month negative-conversion rate.

According to ITT analysis, the 1-month negative-conversion rate was 26% (7/27) in the FMT group and 10% (2/21) in the control group; however, the difference between groups was not statistically significant (*p* = 0.264). The 3-month negative-conversion rate was 52% (14/27) in the FMT group, and 24% (5/21) in the control group, showing a marginally significant difference (*p* = 0.049) (Figure 2). The natural negative-conversion rate of the control group increased over time.

According to the per-protocol analysis, the 1-month negative-conversion rate was 21% (5/24) in the FMT group and 5% (1/19) in the control group, but the difference was not statistically significant (*p* = 0.143). The 3-month negative-conversion rate was 47% (9/19) in the FMT group, and 25% (3/12) in the control group, but the difference was not statistically significant (*p* = 0.274).

In a subgroup ITT analysis, the 1-month negative-conversion rate of CRE was 36% (5/14) in the FMT group and 10% (1/10) in the control group, and that of VRE was 13% (2/15) in the FMT group and 9% (1/11) in the control group. These differences in the CRE and VRE 1-month negative-conversion rates were not significant (*p* = 0.341 and *p* > 0.999, respectively). The 3-month negative-conversion rate of CRE was 71% (10/14) in the FMT group and 30% (3/10) in the control group, and that of VRE was 33% (5/15) in the FMT group and 18% (2/11) in the control group. The differences in the CRE and VRE 3-month negative-conversion rates were not statistically significant (*p* = 0.095 and *p* = 0.658, respectively).

In the subgroup per-protocol analysis, the 1-month negative-conversion rate of CRE was 25% (3/12) in the FMT group and 0% (0/8) in the control group, and that of VRE was 15% (2/13) in the FMT group and 9% (1/11) in the control group. These differences in the CRE and VRE 1-month negative-conversion rates were not statistically significant (*p* = 0.242 and *p* > 0.999, respectively). The 3-month negative conversion rate of CRE was 67% (6/9) in the FMT group and 40% (2/5) in the control group, and that of VRE was 30% (3/10) in the FMT group and 14% (1/7) in the control group. These differences in the CRE and VRE 3-month negative-conversion rates were not statistically significant (*p* = 0.580 and *p* = 0.603, respectively).

**Figure 3:**
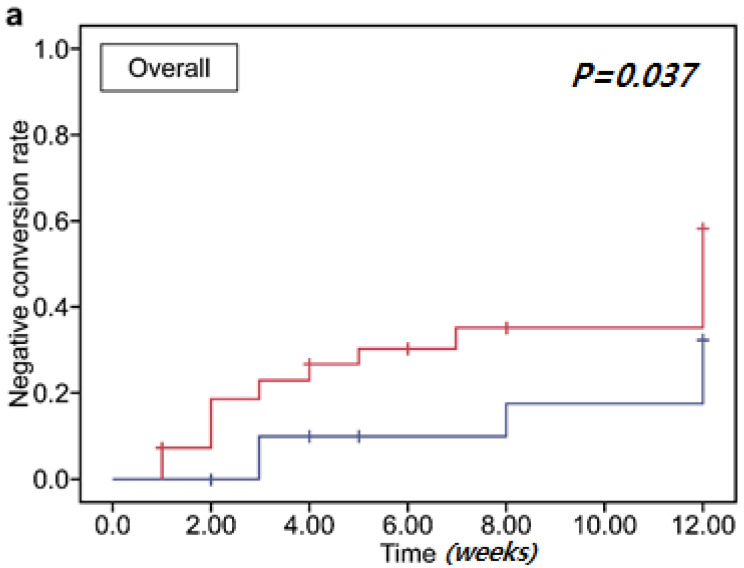

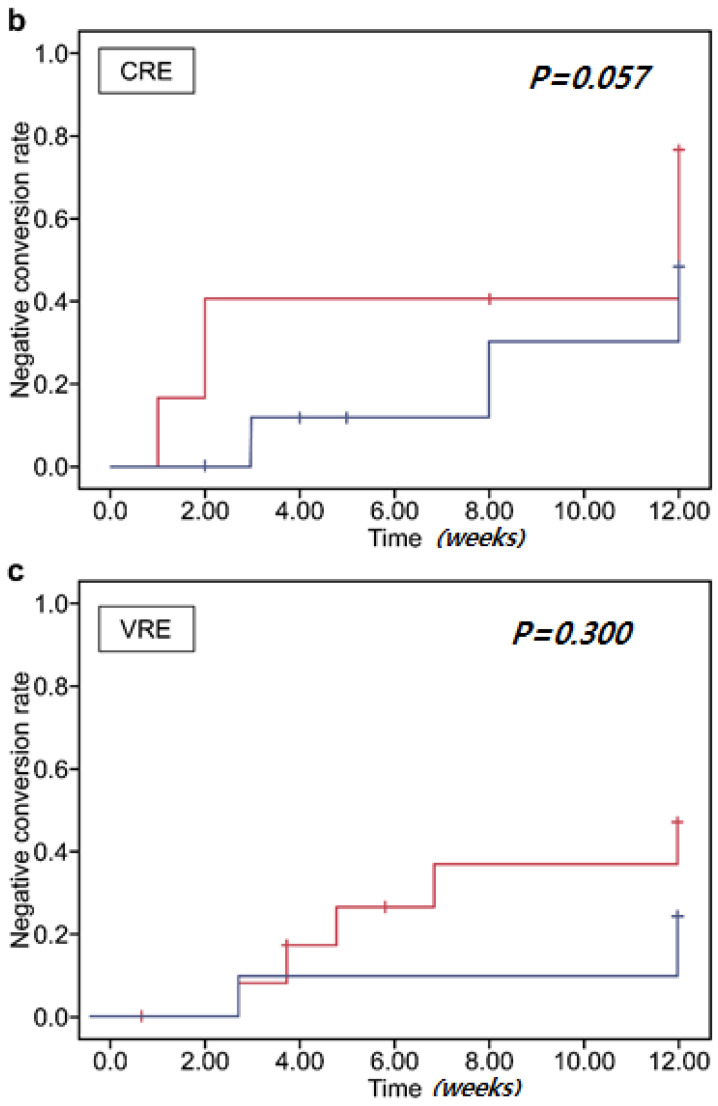
Multidrug-resistant organism (MDRO) clearance rate following fecal microbiota transplantation (FMT). (**a**) Overall cumulative clearance. (**b**) Cumulative clearance of carbapenem-resistant *Enterobacteriaceae.* (**c**) Cumulative clearance of vancomycin-resistant enterococci.

Figure 3 shows the cumulative negative-conversion rates. The overall negative-conversion rate in the FMT group was significantly higher than that in the control group (*p* = 0.037) according to subgroup analysis. The cumulative CRE and VRE negative-conversion rates did not differ significantly by group (*p* = 0.057 and *p* = 0.300, respectively). There was no significant difference in MDRO clearance according to the FMT delivery method (Supplementary Table S3).

### 3.4. Clearance of Multidrug-Resistant Organisms Associated with Antibiotics

We evaluated the effect of antibiotics 1 month before and after FMT or enrollment on the clearance of MDROs. Patients colonized by MDROs tend to have multiple comorbidities and diseases, and they cannot discontinue antibiotic use [29,30]. Various antibiotics were used, including cephalosporins (ceftazidime, ceftriaxone, and cefepime), sulfamethoxazole-trimethoprim, aminoglycosides (amikacin and gentamycin), penicillin (piperacillin/tazobactam and ampicillin/sulbactam), glycopeptides (vancomycin and teicoplanin), and carbapenems (meropenem and ertapenem). The types of antibiotics and the duration of antibiotic use did not differ significantly between groups (Supplementary Table S1). Analyzing antibiotics associated with 1-month and 3-month negative conversion, the duration of antibiotic use before FMT or enrollment had a significant association with 1-month negative conversion (*p* = 0.008) (Supplementary Table S2). In addition, the use of broad-spectrum penicillin after FMT or enrollment and antibiotic use before FMT or enrollment were significantly associated with 3-month negative conversion (*p* = 0.015 and *p* = 0.041, respectively).

### 3.5. Adverse Events

Adverse events, including non-infection fever (5/27, 19%), diarrhea (4/27, 15%), and constipation (1/27, 4%), were observed within 1 week after FMT. These adverse events may have been associated with FMT. Within 1 month, there were three (3/27, 11%) cases of UTI, two cases of pneumonia (2/27, 7%), one case of suspected gout (1/27, 4%), and one case of cholecystitis (1/27, 4%). It is not possible to confirm whether these adverse events were related to FMT, but it is more likely that these events were associated with patient comorbidities. The frequency of these adverse events did not differ significantly according to the FMT delivery method (Supplementary Table S4).

### 3.6. Microbiome Analysis before and after Fecal Microbiota Transplantation

**Figure 4:**
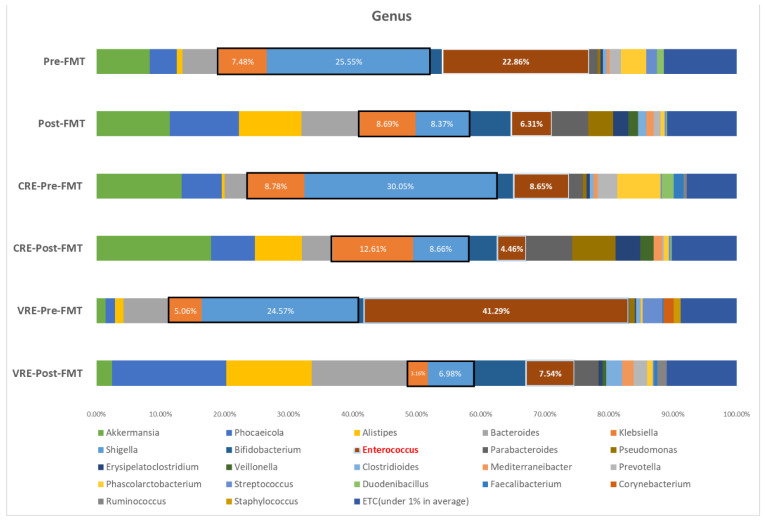
Comparison of abundance according to genus: pre- and post-fecal microbiota transplantation (FMT), pre- and post-FMT in patients infected with carbapenem-resistant *Enterobacteriaceae* (CRE); and pre- and post-FMT in patients infected with vancomycin-resistant enterococci (VRE). Black box indicated *Enterobacteriaceae* family including *Klebsiella* and *shigella*.

We compared paired samples before and 3 weeks after FMT from 15 patients (seven patients infected with VRE and eight infected with CRE). The other samples could not be analyzed owing to DNA damage due to storage. Figure 4 shows the changes in bacterial taxa before and after FMT. There were reductions in the average bacterial compositions, such as the abundance of the *Enterobacteriaceae* family (from 33% to 17%), which is associated with CRE. The proportion of *Enterococcus*, which is associated with VRE, was reduced from 23% to 6%. Comparing the proportion of each genus between patients with CRE and VRE infections before FMT, the proportion of *Enterococcus* was higher in patients infected with VRE than in those infected with CRE (41% vs. 9%), whereas *Klebsiella* (9% vs. 5%) was more prominent in patients infected with CRE than in those infected with VRE.

**Figure 5:**
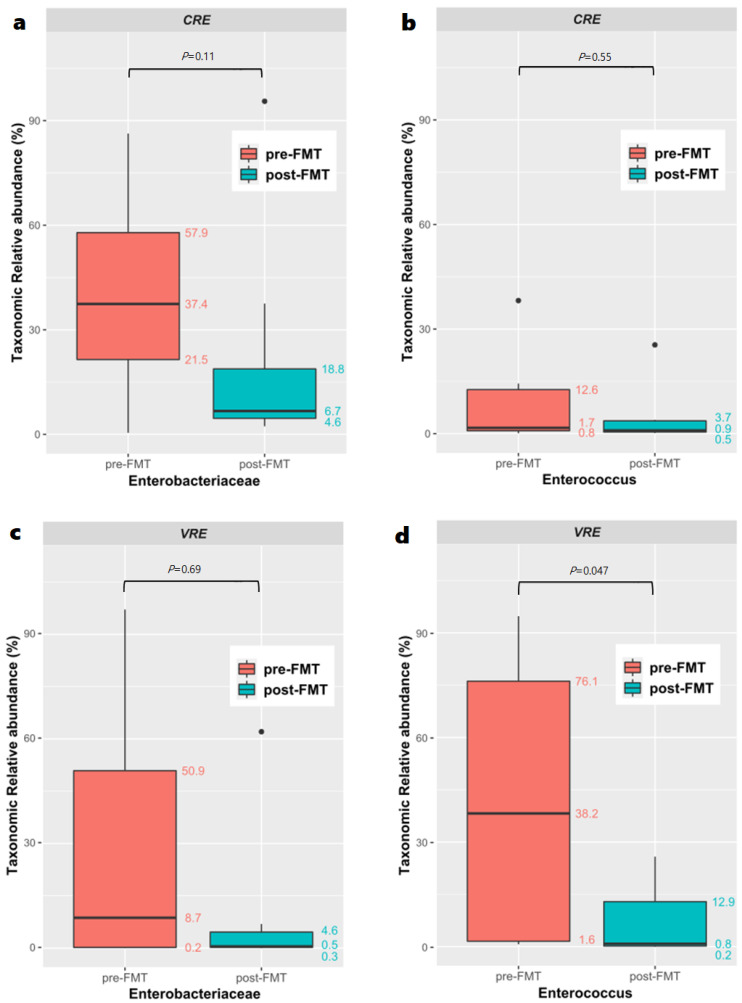
Comparison of the relative abundance of: (**a**) carbapenem-resistant *Enterobacteriaceae* (CRE) in the pre- and post-fecal microbiota transplantation (FMT) groups; (**b**) *Enterococcus* genus CRE in the pre- and post-FMT groups; (**c**) vancomycin-resistant *Enterobacteriaceae* (VRE) in the pre- and post-FMT groups; and (**d**) *Enterococcus* genus VRE in the pre- and post-FMT groups.

The relative abundance of the *Enterobacteriaceae* family and *Enterococcus* genus, which were the main MDROs colonizing the patients with CRE and VRE infections are shown in Figure 5. The abundance of the *Enterobacteriaceae* family (median [IQR]; 37.41 [21.47–57.87] before vs. 6.70 [4.60–18.77] after, *p* = 0.11) in patients infected with CRE changed following FMT. In contrast, the abundance of *Enterococcus* in patients infected with CRE did not change after FMT (median [IQR]: 1.66 [0.83–12.63] before vs. 0.92 [0.46–3.66] after, *p* = 0.55). However, the abundance of *Enterococcus* in patients infected with VRE significantly changed after FMT (median [IQR]: 38.18 [1.58–76.11] before vs. 0.84 [0.18–12.9] after, *p* = 0.047). In contrast, the abundance of *Enterobacteriaceae* (median [IQR]: 8.74 [0.22–50.88] before vs. 0.52 [0.26–4.63] after, *p* = 0.886) in patients infected with VRE remained unchanged following FMT.

**Figure 6:**
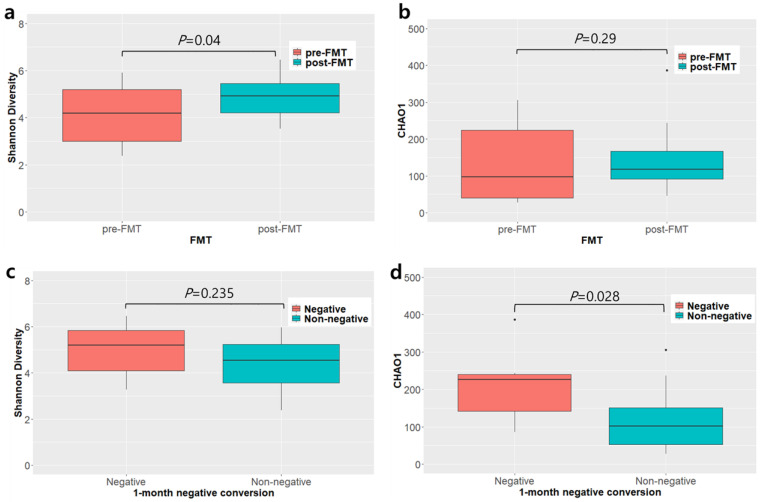
Difference in alpha-diversity. (**a**) Shannon index diversity of the pre- and post-fecal microbiota transplantation (FMT) groups. (**b**) Chao1 diversity of the pre- and post-FMT groups. (**c**) Shannon index diversity of the 1-month negative-conversion and 1-month non-negative-conversion groups. (**d**) Chao1 diversity of the 1-month negative-conversion and 1-month non-negative-conversion groups.

Alpha-diversity indices, including diversity index (Shannon) and species richness estimate (Chao1), were different between pre- and post-FMT groups (*p* = 0.04 and *p* = 0.29, respectively; Figure 6a,b). We divided the patients into two groups: those who achieved decolonization within 1 month of FMT (three patients and six cases; 1-month positive) and those who exhibited consistent colonization within 1 month after FMT (12 patients and 24 cases; 1-month negative). Species diversity (Chao1) in the 1-month negative-conversion group was significantly higher than that in the 1-month non-negative-conversion group (*p* = 0.028). Nevertheless, the Shannon index was not significantly different between groups (Figure 6c,d). Beta-diversity between groups differed but not significantly (*p* = 0.098, PERMANOVA) (Supplementary Figure S1).

There were significant differences in the specific taxa between groups (Supplementary Figure S2). Phylum Euryarchaeota, class Bacteroidia, class Methanobacteria orders Bacteroidales, Veillonellales, and Methanobacteriales; families *Methanobacteriaceae* and *Veillonellaceae*; genus *Methanobrevibacter*; and species *Bacteroides finegoldii, Methanobrevibacter smithii*, and *Veillonella dispar*, were significantly more abundant in the post-FMT group. We also compared the microbiomes between the 1-month negative-conversion and 1-month non-negative-conversion groups (Supplementary Figure S3). Order *Selenomonadales*; families *Prevotellaceae* and *Selenomonadaceae*; genera *Prevotella*, *Megamonas*, *Ocillibacter*, *Facalibacterium*, *Gemminger*, *Ethanoligenens*, *Ruminococcus*, *Roseburia*, *Dorea*, *Flintibacter*, and *Lactobacillus*, and several species, were more abundant in the 1-month negative-conversion group.

## 4. Discussion

To our knowledge, this study is the first prospective case–control study to compare the clearance of MDROs, including VRE and CRE, between the FMT and control groups in patients with multiple comorbidities via microbiome analysis. The patients in the FMT group were significantly younger than the patients in the control group. However, age was not independently associated with the clearance of MDROs. The overall 1-month clearance rate of CRE or VRE in the FMT group was not significantly better than that in the control group, regardless of antibiotic use before and after FMT. The overall 3-month clearance of MDROs in the FMT group was marginally higher than that in the control group. The overall cumulative negative-conversion rate in the FMT group was also significant. In subgroup analysis, CRE tended to be decolonized more effectively than VRE in our study. There were no presumed fatal adverse events associated with FMT. In microbiome analysis, the abundance of the dominant MDRO family or genus in stool, such as *Enterobacteriaceae* or *Enterococcus*, was reduced. In particular, the difference in the abundance of *Enterococcus* in patients infected with VRE was significantly decreased. These findings suggest that FMT is a potential strategy for eliminating intestinal colonization by MDROs.

Our study did not achieve the primary endpoint (1-month negative conversion). Various factors, such as antibiotic use, might affect MDRO negative conversion. We observed that the use of antibiotics and broad-spectrum penicillin before FMT were significantly associated with a weaker decolonization effect of FMT. There are limited data available on FMT failure for clearance of MDRO. However, systemic antibiotics are known to increase the risk of FMT failure and *C. difficile* infection [31]; therefore, prior antibiotic use within the previous month and broad-spectrum antibiotic use were considered before FMT.

Similarly, previous studies have shown that FMT can eradicate MDROs, including CRE, VRE, and extended-spectrum beta-lactamase producers [9,10,13,32], with a clearance rate of 37.5–87.5% [13]. The clearance of CRE by FMT was reported to be effective, which is consistent with our current findings [11,33]. Previous reports have also revealed adverse events associated with FMT for MDROs [34]. The frequency of adverse events related to FMT was approximately 19–44%, and serious adverse events constituted approximately 1–6% [34,35,36], similar to those in our study. Serious events might have occurred following FMT in our study. Moreover, patients with CRE or VRE infections generally have multiple comorbidities, resulting in a high probability of side effects. Several patients in the control group died owing to underlying diseases. However, it is difficult to conclude whether these side effects were associated with FMT. The FMT delivery method suitable for the patient’s condition was selected, taking their multiple comorbidities into account. There was no difference in MDRO clearance according to the FMT delivery method in our study. MDRO decolonization according to the delivery method in MDRO FMT has not been well-studied. A recent meta-analysis showed no difference in MDRO clearance between FMT delivery via the upper route and that via the lower route [11]. Colonoscopy is considered superior to other methods for FMT delivery in *C. difficile* infection, so future research is needed [37].

In this study, VRE showed a lower decolonization rate than CRE. The comparison between the clearance of CRE and VRE by FMT is controversial. Previous studies showed a CRE clearance rate similar to that in our study [10,11,13]. In contrast, other studies reported that the clearance rate of VRE was lower than that of CRE [10,18]. A meta-analysis showed that among MDROs, VRE showed the lowest clearance rate following FMT [11]. VRE clearance was approximately 60% at 1 month and only 40–50% at 3 months [10,12,18]. In other studies, the period in which approximately 50% of CRE was eradicated was approximately 12 weeks, whereas VRE decolonization took approximately 25 weeks [27,28]. The reason for the poor clearance of VRE is that VRE is associated with dysbiosis and inflamed colons in patients. It is thought that the clearance rate is determined by the abundance of MDROs before FMT. *Klebsiella* was rapidly removed in patients infected with CRE, as indicated by its relatively low abundance (9%). However, as the abundance of *Enterococcus* in patients infected with VRE was high (41%), it would be difficult to eliminate VRE to an undetectable threshold with only one instance of FMT. Therefore, repeated FMT is considered to be more efficient for eradicating MDROs [14].

We hypothesized that dysbiosis is the main reason for MDRO colonization based on a previous study [27] in which the *Enterobacteriaceae* family was dominant in patients infected with CRE prior to FMT, and the *Enterococcus* genus was dominant in the feces of patients infected with VRE before FMT. Prolonged antibiotic use and hospitalization are known as the main causes of MDRO colonization [27,28,38]. The patients enrolled in this study had prior risk factors of MDRO colonization, such as multiple comorbidities, prolonged antibiotic use, and long stays at a long-term care facility before admission [39].

In line with our findings, a previous study revealed that *Enterococcus* played a major role in dysbiosis in VRE-colonized patients [40]. CRE carriers also exhibited lower phylogenetic diversity and abundance of dysbiotic microbiota, which were enriched with members of the *Enterobacteriaceae* family [41]. FMT was shown to be an effective strategy to alleviate dysbiosis in MDRO colonization. The total abundance of *Enterococcus* and *Enterobacteriaceae* members was reduced after FMT. In addition, the abundance of the members of the order *Bacteroidales*, recognized as important bacteria, also increased after FMT, as shown in other studies, as it decreased dysbiosis in MDRO-colonized patients [17,27,39,40,41,42]. It was difficult to find a reference in the literature for the other taxa [17,40,41]. This may be owing to differences in the bacterial composition of VRE and CRE in pre-FMT patients or may be caused by the bacterial diversity in the donor. In the present study, *Bacteroidia* (3rd), *Veillonellales* (9th), *Bacteroidales* (2nd), *Veillonellaceae* (17th), *Bacteroidaceae* (2nd), and *Bacteroides* (1st) were the main bacterial taxa in the donor. We also compared bacterial diversity between positive responders within 1 month and negative responders within 1 month. Species richness, an indicator of diversity, was a predictive factor for MDRO decolonization. The other previous studies showed that poor responders to FMT had low bacterial diversity, which is consistent with our study findings [17,18]. *Prevotellaceae* was dominant in the 1-month negative-conversion group. Some studies showed that the efficacy of FMT is based on certain genera, such as *Ruminococcaceae*, *Lachnospiraceae*, and *Prevotellaceae* [43]; therefore, we hypothesized that MDRO decolonization would be high if FMT was performed effectively. On the basis of these findings, we can assume that the burden of dysbiosis and the abundance of MDROs are correlated to FMT. Therefore, the strategy of alleviating dysbiosis before FMT by applying probiotics and de-escalating antibiotics may enhance the efficacy of FMT for MDRO decolonization [44,45].

The strengths of our study are as follows. First, we compared FMT invention groups with a control group. To our knowledge, despite being a single-center study, this study is the largest study of FMT reported to date [9,11]. Second, we revealed the mechanism underlying MDRO eradication by microbiome analysis of fecal samples from patients. FMT was effective in reversing dysbiosis, as MDRO colonization was increased in feces, thereby decreasing the abundance of MDROs. We confirmed that the abundance of *Enterobacteriaceae* and *Enterococcus* decreased in patients with CRE and VRE infections and that treatment of dysbiosis is the key factor for clearance of MDROs.

There are several limitations to this study. First, as this was not a randomized controlled study, the statistical power was low. Second, the number of enrolled patients was insufficient to show a significant difference in MDRO decolonization between groups. More patients than expected were lost to follow-up in the FMT and control groups owing to multiple comorbidities. We used an ITT analysis to account for participant loss to follow-up. Significant cumulative negative conversion of FMT and the microbiome analysis results in our study suggest that FMT might be effective for clearance of MDRO. Third, we could not analyze the entire sample in the intervention and control groups. Therefore, the relative abundance of the key taxa in the enrolled patients did not show a significant difference before and after FMT.

## 5. Conclusions

FMT may be an effective strategy for MDRO decolonization in the intestinal tract. Microbiome analysis supported that FMT was effective for MDRO decolonization by reversing dysbiosis. However, older patients and patients with multiple comorbidities need close monitoring for a period after FMT. It is unknown whether the side effects in these patients are related to FMT; further research on this subject is warranted. Moreover, our current results should be confirmed by larger randomized controlled trials.

## Figures and Tables

**Table 1:** Comparison of the baseline clinical characteristics of the enrolled patients between the FMT and control groups.

	FMT Group (*N* = 27)	Control Group (*N* = 21)	*p*-Value
**Age, year, median (IQR)**	73 (63–81)	80 (73–84)	0.03 *
**Sex**			0.422
Male	11 (41)	11 (52)	
Female	16 (59)	10 (48)	
**Multidrug-resistant organism**			0.444
CRE	12 (44)	10 (48)	
VRE	13 (48)	11 (52)	
CRE + VRE	2 (7)	0 (0)	
**Disease (at admission)**			
Pneumonia	12 (44)	13 (62)	0.23
Urinary tract infection	12 (44)	7 (33)	0.435
*Clostridium difficile* colitis	11 (41)	7 (33)	0.599
**Comorbidity**			
Diabetes			0.922
Uncomplicated	5 (19)	3 (14)	
Complicated	7 (26)	6 (29)	
Liver disease			0.507
Mild	0 (0)	1 (5)	
Moderate to severe	1 (4)	1 (5)	
Malignancy	1 (4)	3 (14)	0.188
Chronic kidney disease	3 (11)	1 (5)	0.43
Congestive heart failure	9 (33)	6 (29)	0.724
Peripheral arterial disease	1 (4)	0 (0)	0.373
Chronic obstructive pulmonary disease	5 (19)	8 (38)	0.13
Cerebral vascular accident	7 (26)	8 (38)	0.367
Hemiplegia	10 (37)	9 (43)	0.683
Rheumatic disease	1 (4)	1 (5)	0.856
Dementia	5 (19)	3 (14)	0.696
Peptic ulcer	2 (7)	1 (5)	0.707
Charlson Comorbidity Index, median (IQR)	2 (1–5)	3 (2–5)	0.378
**FMT delivery method**			
Colonoscopy	14 (52)	···	
Duodenoscopy	1 (4)	···	
Duodenoscopy + colonoscopy	12 (44)	···	
**Bowel preparation**	10 (42)	···	

Abbreviations: CRE, carbapenem-resistant *Enterobacteriaceae*; FMT, fecal microbiota transplantation; IQR, interquartile range; VRE, vancomycin-resistance enterococci. * *p* < 0.05.

## Data Availability

The dataset presented in this study is available from the corresponding author upon reasonable request.

## Supplementary Materials

The following supporting information can be downloaded at: https://www.mdpi.com/article/10.3390/biomedicines10102404/s1, Figure S1: Taxonomic cladogram obtained using linear discriminant analysis effect size (LEfSe) analysis of the 16S sequences; Figure S2: Difference in beta-diversity pre- and post-fecal microbiota transplantation (FMT) estimated as (a) Unweighted UniFrac (b) Bray–Curtis dissimilarity index between the pre- and post-FMT group; Figure S3: Taxonomic linear discriminant analysis effect size (LEfSe) analysis of the 16S sequences with the greatest differences in abundance between the 1-month negative-conversion and 1-month non-negative-conversion groups; Table S1: Comparison of antibiotic use between the fecal microbiota transplantation and control groups; Table S2: Analysis of age and antibiotic use among study participants associated with 1-month negative conversion and 3-month negative conversion; Table S3: Analysis of age and fecal microbiota transplantation method for enrolled patients associated with 1-month negative conversion and 3-month negative conversion.
