# Supplementary material for: Efficacy and Safety of Fecal Microbiota Transplantation for Clearance of Multidrug-Resistant Organisms under Multiple Comorbidities: A Prospective Comparative Trial
Source: Biomedicines. 2022 Sep 26;10(10):2404. doi: 10.3390/biomedicines10102404 (PMC9598999; doi:10.3390/biomedicines10102404)
Supplement: Supplementary file 1 [file biomedicines-10-02404-s001.zip › Supplementary_table_S1.pdf]

Supplementary Table S1. Comparison of antibiotic use between the fecal microbiota transplantation and control groups

Variable	FMT group (N=27)	Control group (N=21)	*P*-value
**Antibiotic use after FMT or enrolment (>7 days), n (%)**	16 (59.3)	10 (47.6)	0.609
Cephalosporin	4 (14.8)	5 (23.8)	0.675
Sulfamethoxazole-trimethoprim	3 (11.1)	2 (9.5)	>0.999
Aminoglycoside	3 (11.1)	0 (0.0)	0.329
Broad-spectrum penicillin	6 (22.2)	7 (33.3)	0.595
Quinolone	6 (22.2)	2 (9.5)	0.435
**Antibiotic use after FMT or enrolment (≤7 days), n (%)**	15 (55.6)	16 (76.2)	0.239
Cephalosporin	3 (11.1)	4 (19.0)	0.718
Sulfamethoxazole-trimethoprim	2 (7.4)	0 (0.0)	0.585
Aminoglycoside	2 (7.4)	2 (9.5)	>0.999
Broad-spectrum penicillin	3 (11.1)	5 (23.8)	0.435
Quinolone	2 (7.4)	3 (14.3)	0.766
Glycopeptide	3 (11.1)	2 (9.5)	>0.999
Oral vancomycin	2 (7.4)	1 (4.8)	>0.999
Carbapenem	1 (3.7)	0 (0.0)	>0.999
**Antibiotic use before FMT or enrolment, n (%)**	23 (85)	21 (100.0)	0.188

**Variable**	**FMT group (N=27)**	**Control group (N=21)**	***P*-value**
Cephalosporin	16 (59)	6 (28.6)	0.068
Sulfamethoxazole-trimethoprim	2 (7.4)	2 (9.5)	>0.999
Aminoglycoside	3 (11.1)	5 (23.8)	0.435
Broad-spectrum penicillin	14 (51.9)	9 (42.9)	0.743
Quinolone	11 (40.7)	8 (38.1)	>0.999
Glycopeptide	6 (22.2)	4 (19.0)	>0.999
Oral vancomycin	4 (14.8)	2 (9.5)	0.912
Tigecycline	1 (3.7)	0 (0.0)	>0.999
Carbapenem	4 (14.8)	2 (9.5)	0.912
**Total antibiotic treatment duration (mean** ± SD)	24.8 ± 14.7	24.2 ± 13.9	0.898
**Antibiotic treatment duration before FMT or enrolment (median, IQR)**	18.0 [5.0;30.0]	28.0 [14.0;32.0]	0.051
**Antibiotic treatment duration after FMT or enrolment (median, IQR)**	3.0 [0.0;22.0]	0.0 [0.0; 2.0]	0.061

Abbreviation: FMT, fecal microbiota transplantation; IQR, interquartile range; SD, standard deviation
