# Supplementary material for: Efficacy and Safety of Fecal Microbiota Transplantation for Clearance of Multidrug-Resistant Organisms under Multiple Comorbidities: A Prospective Comparative Trial
Source: Biomedicines. 2022 Sep 26;10(10):2404. doi: 10.3390/biomedicines10102404 (PMC9598999; doi:10.3390/biomedicines10102404)
Supplement: Supplementary file 1 [file biomedicines-10-02404-s001.zip › Supplementary_table_S2.pdf]

Supplementary Table S2. Analysis of age and antibiotic use among study participants associated with 1-month negative conversion and 3-month negative conversion.

**Variable**	**1-month negative conversion**			**3-month negative conversion**		
	No (n=39)	Yes (n=9)	*P*-value	No (n=29)	Yes (n=19)	*P*-value
Age, median [IQR], years	78 [70–82.5]	67 [61–81]	0.328	78 [70–83]	70 [62–81.5]	0.187
Antibiotic use after FMT or enrolment (>7 days)	23 (59.0)	3 (33.3)	0.307	17 (58.6)	9 (47.4)	0.639
Cephalosporin	8 (20.5)	1 (11.1)	0.859	6 (20.7)	3 (15.8)	0.962
Sulfamethoxazole-trimethoprim	4 (10.3)	1 (11.1)	>0.999	3 (10.3)	2 (10.5)	>0.999
Aminoglycoside	2 (5.1)	1 (11.1)	>0.999	1 (3.4)	2 (10.5)	0.703
Broad-spectrum penicillin	12 (30.8)	1 (11.1)	0.435	12 (41.4)	1 (5.3)	**0.015**
Quinolone	7 (17.9)	1 (11.1)	>0.999	6 (20.7)	2 (10.5)	0.598
Antibiotic use after FMT or enrolment (≤7 days)	26 (66.7)	5 (55.6)	0.809	20 (69.0)	11 (57.9)	0.634
Cephalosporin	6 (15.4)	1 (11.1)	>0.999	5 (17.2)	2 (10.5)	0.821
Sulfamethoxazole-trimethoprim	2 (5.1)	0 (0.0)	>0.999	1 (3.4)	1 (5.3)	>0.999

Variable	1-month negative conversion			3-month negative conversion		
Aminoglycoside	3 (7.7)	1 (11.1)	>0.999	2 (6.9)	2 (10.5)	>0.999
Broad-spectrum penicillin	7 (17.9)	1 (11.1)	>0.999	5 (17.2)	3 (15.8)	>0.999
Quinolone	5 (12.8)	0 (0.0)	0.596	4 (13.8)	1 (5.3)	0.643
Glycopeptide	3 (7.7)	2 (22.2)	0.496	3 (10.3)	2 (10.5)	>0.999
Oral vancomycin	2 (5.1)	1 (11.1)	>0.999	2 (6.9)	1 (5.3)	>0.999
Carbapenem	1 (2.6)	0 (0.0)	>0.999	0 (0.0)	1 (5.3)	0.83
Antibiotic usage before FMT or enrolment	37 (94.9)	7 (77.8)	0.316	29 (100.0)	15 (78.9)	**0.041**
Cephalosporin	20 (51.3)	2 (22.2)	0.228	14 (48.3)	8 (42.1)	0.902
Sulfamethoxazole-trimethoprim	4 (10.3)	0 (0.0)	0.738	3 (10.3)	1 (5.3)	0.929
Aminoglycoside	7 (17.9)	1 (11.1)	>0.999	6 (20.7)	2 (10.5)	0.598
Broad-spectrum penicillin	22 (56.4)	3 (33.3)	0.379	17 (58.6)	8 (42.1)	0.41
Quinolone	17 (43.6)	2 (22.2)	0.422	13 (44.8)	6 (31.6)	0.538
Glycopeptide	7 (17.9)	3 (33.3)	0.569	5 (17.2)	5 (26.3)	0.694
Oral vancomycin	4 (10.3)	2 (22.2)	0.675	3 (10.3)	3 (15.8)	0.911
Tigecycline	1 (2.6)	0 (0.0)	>0.999	0 (0.0)	1 (5.3)	0.83

Variable	1-month negative conversion			3-month negative conversion		
Carbapenem	6 (15.4)	0 (0.0)	0.485	3 (10.3)	3 (15.8)	0.911
Total antibiotic treatment duration, median (IQR), days	30 [15–34]	15 [5–22]	0.128	26 [15–34]	22 [11.5–35.5]	0.712
Antibiotic treatment duration before FMT or enrollment, median (IQR), days	25 [14–31]	5 [3–12]	**0.008**	23 [14–31]	12 [4–30]	0.073
Antibiotic treatment duration after FMT or enrollment, median (IQR), days	0 [0–7]	0 [0–23]	0.708	0 [0–5]	1 [0–22]	0.223

Abbreviations: FMT, fecal microbiota transplantation; IQR, interquartile range
