# Supplementary material for: Efficacy and Safety of Fecal Microbiota Transplantation for Clearance of Multidrug-Resistant Organisms under Multiple Comorbidities: A Prospective Comparative Trial
Source: Biomedicines. 2022 Sep 26;10(10):2404. doi: 10.3390/biomedicines10102404 (PMC9598999; doi:10.3390/biomedicines10102404)
Supplement: Supplementary file 1 [file biomedicines-10-02404-s001.zip › Supplementary_table_S3.pdf]

Supplementary Table S3. Analysis of age and fecal microbiota transplantation method for enrolled patients associated with 1-month negative conversion and 3-month negative conversion.

	1-month negative conversion			3-month negative conversion		
	Yes (n=7)	No (n=20)	*P-value*	Yes (n=14)	No (n=13)	*P-value*
Age, median [IQR], years	67 [61–75.5]	74.5 [64.5–81]	0.678	67 [55–80]	77 [70–82]	0.224
Bowel preparation	2 (28.6)	9 (45.0)	0.753	4 (28.6)	7 (53.8)	0.345
FMT delivery method			1			0.558
Colonoscopy	4 (57.1)	10 (50.0)		6 (42.9)	8 (61.5)	
Duodenoscopy ± Colonoscopy	3 (42.9)	10 (50.0)		8 (57.1)	5 (38.5)	

Abbreviation: FMT, fecal microbiota transplantation; IQR, interquartile range
