# Supplementary figures and images for: Efficacy and Safety of Fecal Microbiota Transplantation for Clearance of Multidrug-Resistant Organisms under Multiple Comorbidities: A Prospective Comparative Trial
Source: Biomedicines. 2022 Sep 26;10(10):2404. doi: 10.3390/biomedicines10102404 (PMC9598999; doi:10.3390/biomedicines10102404)

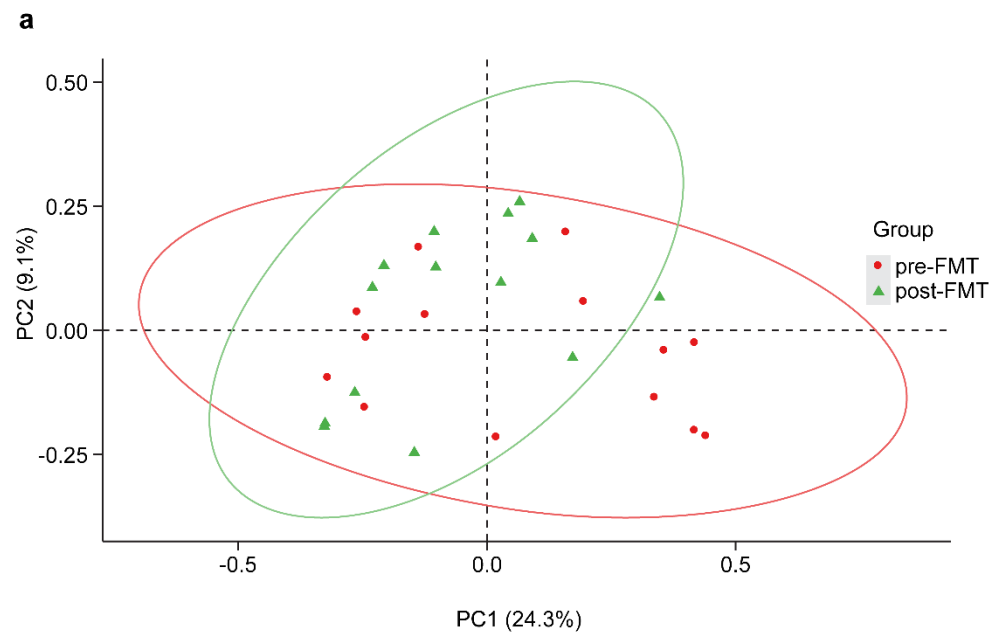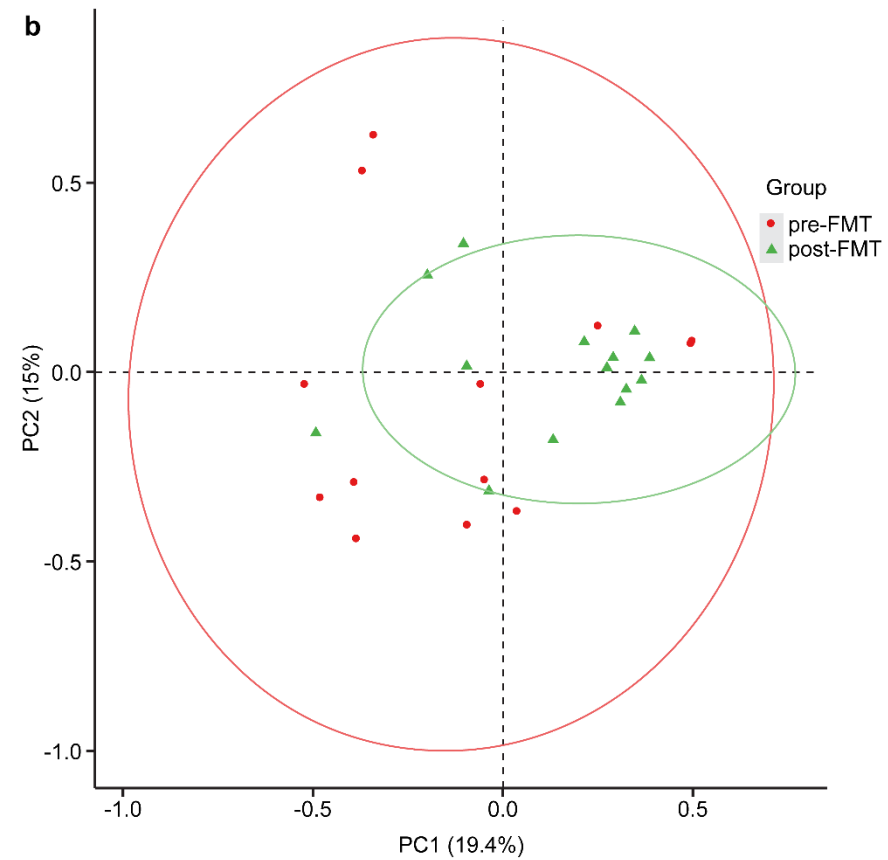

**Supplementary Figure S1**

Supplement: Supplementary file 1 [file biomedicines-10-02404-s001.zip › Supplementary_Figure_S1.pdf]

**a**

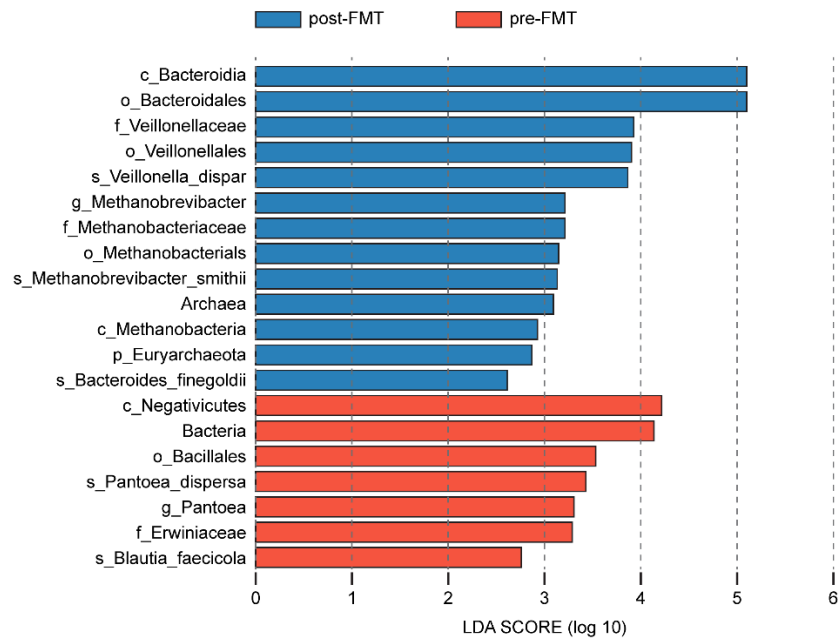

**b**

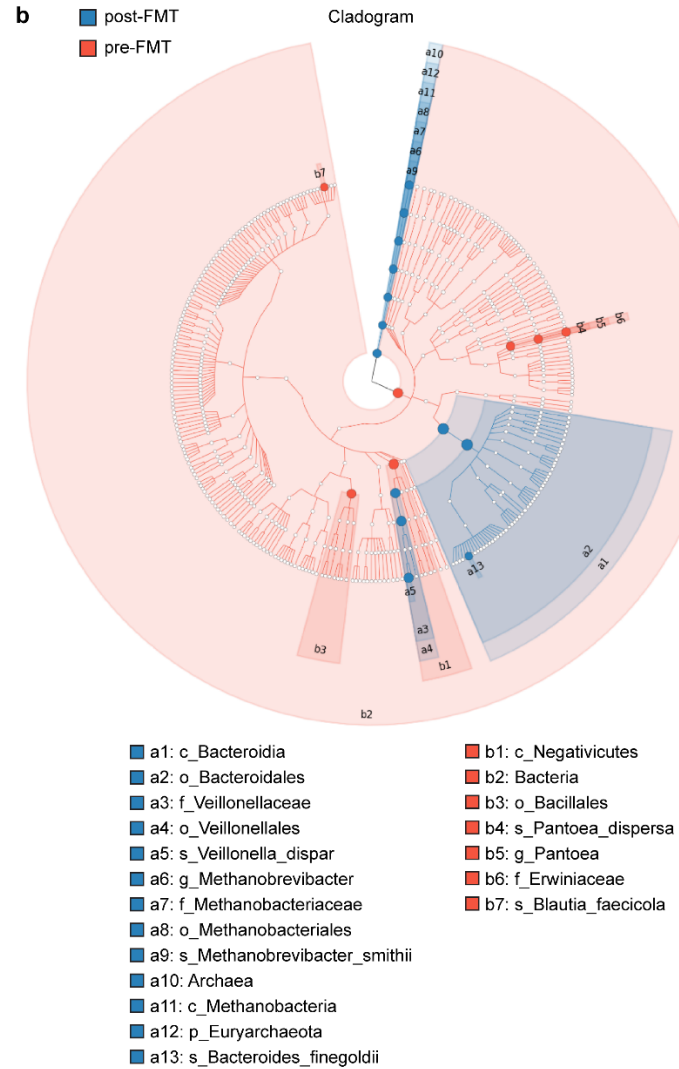

**Supplementary Figure S2**

Supplement: Supplementary file 1 [file biomedicines-10-02404-s001.zip › Supplementary_Figure_S2.pdf]

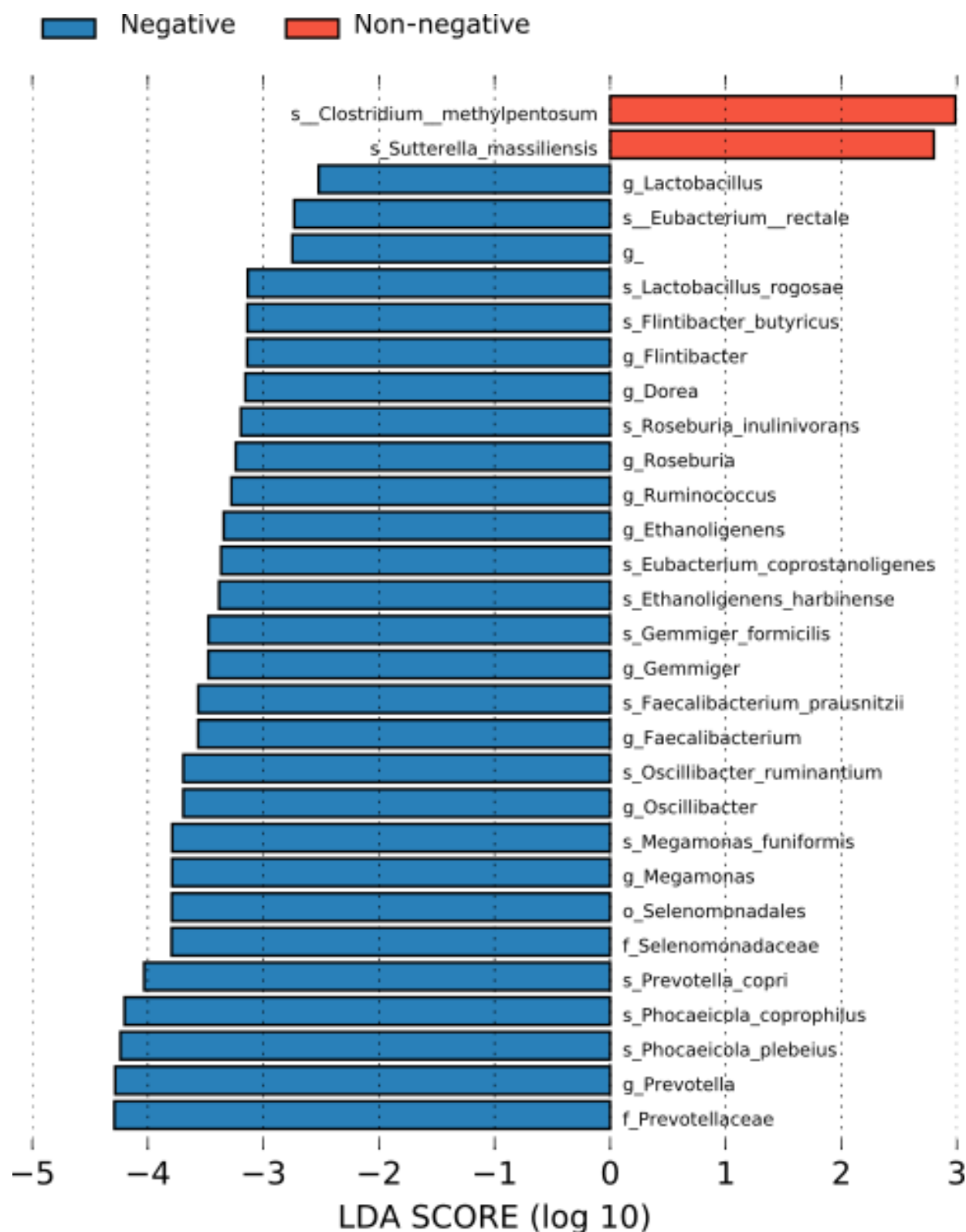

**Supplementary Figure S3**

Supplement: Supplementary file 1 [file biomedicines-10-02404-s001.zip › Supplementary_Figure_S3.pdf]
